# Measurement of energy availability in highly trained male endurance athletes and examination of its associations with bone health and endocrine function

**DOI:** 10.1007/s00394-024-03433-8

**Published:** 2024-07-02

**Authors:** Amy McGuire, Giles Warrington, Adam Walsh, Thomas Byrne, Lorna Doyle

**Affiliations:** 1 Department of Sport and Early Childhood Studies, Technological University of the Shannon, Limerick, Ireland; 2 ACTIVE Research Group, Technological University of the Shannon, Limerick, Ireland; 3 Department of Physical Education and Social Sciences, University of Limerick, Limerick, Ireland; 4 Sport and Human Performance Research Centre, Health Research Institute, University of Limerick, Limerick, Ireland; 5 Department of Sport and Exercise Science, South East Technological University, Cork Road, Waterford, Ireland

**Keywords:** Energy availability, Endocrine function, Bone metabolism

## Abstract

**Purpose:**

Despite the introduction of Relative Energy Deficiency in Sport (RED-s) in 2014, there is evidence to suggest that male endurance athletes still present with a high prevalence of low energy availability (LEA). Previous findings suggest that energy availability (EA) status is strongly correlated with impairments in endocrine function such as reduced leptin, triiodothyronine (T_3)_, and insulin, and elevated bone loss. This study aimed to report the current EA status, endocrine function and bone health of highly trained Irish male endurance athletes.

**Methods:**

In this cross-sectional study, participants (*n* = 3 triathletes; *n* = 10 runners) completed a 7-day testing period during the competition season using lab-based measures, to ascertain EA status, hormone level and rates of bone metabolism. Serum blood samples were obtained to assess hormone levels and markers of bone metabolism.

**Results:**

Mean EA was < 30 kcal/kg lean body mass (LBM)/day in 76.9% of athletes. There was a strong association between LEA and low carbohydrate intake, and lower LBM. Mean levels of insulin, IGF-1 and leptin were significantly lower than their reference ranges. Elevated mean concentrations of β-CTX and a mean P1NP: β-CTX ratio < 100, indicated a state of bone resorption.

**Conclusion:**

The EA level, carbohydrate intake, hormone status and bone metabolism status of highly trained male endurance athletes are a concern. Based on the findings of this study, more frequent assessment of EA across a season is recommended to monitor the status of male endurance athletes, in conjunction with nutritional education specific to EA and the associated risks.

## Introduction

Energy availability (EA) is defined as the energy remaining for optimal physiological function after the exercise energy expenditure (EEE) has been subtracted from energy intake (EI) [1]

There is currently no established value for optimal physiological function in male athletes, however, for recreationally active males, 40 kcal/kg lean body mass (LBM)/day appears to be the threshold [2]. Low energy availability (LEA ≤ 30 kcal/kg LBM/day) has shown to lead to a syndrome known as Relative Energy Deficiency in Sport (RED-s), manifesting in conditions such as impaired reproductive functioning, bone health and metabolic health in females, however the physiological effects of LEA in males remains relatively unknown [3, 4]. Endurance exercise utilises high volumes of energy and it is therefore recommended that endurance athletes avoid prolonged periods of LEA in favour of efficiently replenishing energy stores and optimising recovery [5]. Demanding competitive schedules coupled with high volumes of training may render it difficult for endurance athletes to consistently meet their individual energy requirements, resulting in multiple periods of LEA [6]. Recent research has suggested that LEA is prevalent in male endurance athletes such as runners, cyclists and swimmers [7, 8]. However, there is currently a dearth of scientific studies investigating the associations of LEA in male endurance athletes [9]. Hormonal disturbances, including reduced testosterone [10], decreases in leptin [11] and decreased concentrations of insulin [2] are among some of the consequences of LEA. However, conclusive effects of LEA on hormonal status and bone metabolism in male endurance athletes have been difficult to determine due to different methodologies used across studies [7, 12]. Additionally, evidence to support the actual cause of these negative consequences is equivocal [13].

Some research in weight category male athletes and healthy active matched controls suggests that energy deficiency may be responsible for impaired bone health and hormonal disturbances, and not participation in endurance activity [14–16]. In support of these findings, a recent review investigating the effects of LEA on markers of bone metabolism in endurance athletes suggested that procollagen type 1 N-terminal propeptide (P1NP), a marker of bone formation, and cross-linked C-telopeptide of type I collagen (β-CTX), a marker of bone resorption, may be negatively impacted by LEA and not exercise, in both males and females, but females being more sensitive [17]. Conversely, a systematic review by Nagle and Brooks [18], failed to establish negative energy balance as the instigator of impaired bone health in endurance athletes, suggesting that the high volume, inadequate bone loading nature of the cyclical exercise itself was culpable. Similarly, it has been suggested that chronic endurance training may be associated with reduced testosterone levels as the pineal gland, adrenal glands and testes may become exhausted after prolonged exercise, although the mechanism for this action is unknown [19]. This reduction in testosterone could be a symptom of the exercise hypogonadal male condition, as opposed to being a consequence of LEA [20]. These hypotheses support an earlier theory by Bilanin et al., [21] who proposed that decreased bone mass may be due to changes in hormone levels such as increased cortisol and decreased testosterone, which can occur because of prolonged endurance training. Elevated cortisol blocks calcium absorption needed for bone cell growth leading to increased bone resorption and ultimately a reduction in bone mineral density (BMD) [22]. Moreover, adequate levels of testosterone may be necessary for the intestinal absorption of calcium [23] and the stimulation of bone formation [24], in particular the upregulation of osteoblasts. Due to the unequivocal evidence pertaining to the relationship between low energy availability, hormones and bone health, further research is needed to ascertain whether associations exist.

The aim of this research was to assess the incidence of EA, hormone levels and bone metabolism and did not address the question of whether the endurance training itself causes a change in bone metabolism or hormone levels, since each athlete had similar training load with regards to training volume and intensity [2]. This paper adds to the need for research into the physiological effects of RED-s, as discussed in the most recent International Olympic Committee consensus statement on RED-s [25].

## Methods

### Participants

Sixteen Irish male highly trained [26], long distance endurance athletes (n = 3 triathletes, n = 13 runners), (34 ± 5 years; 176 ± 6 cm; 76 ± 12 kg), who trained > 6 h per week [27] were recruited for this study through email, social media and word of mouth, during the competition phase of their respective season. A power analysis was carried out using G*Power software (version 3.1.9.6; Heinrich Heine University, Dusseldorf, Germany), with an effect size of 0.8, an α level of 0.05, and statistical power of 0.8. The required sample size was calculated to be 15 participants. Participants were informed of the purpose and any risks associated with taking part in the investigation before written consent was obtained. Highly trained athletes are defined as competing at the national level [26]. The study was approved by the South East Technological University Ethics Committee (17/HSES/03).

### Assessment of EA

**Fig. 1:**
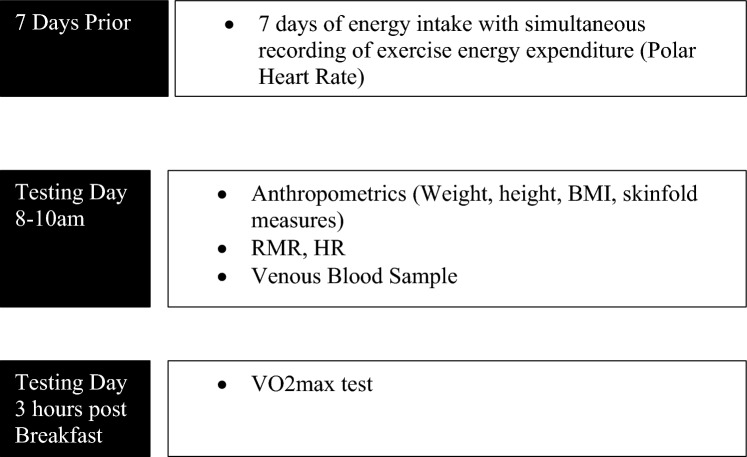
Schematic representation of study design

Figure 1 represent a schematic of the study design. EA was calculated as energy intake (EI) minus calories expended during exercise energy expenditure (EEE), adjusted for resting metabolic rate (RMR) and normalised to lean body mass (LBM) [28].

$${\text{EA}} = {\text{EI}}-{\text{EEE}}\left( {\text{adjusted for RMR}} \right)/\left( {{\text{LBM}}}\right), {\text{ with a unit of kcals}}/{\text{kg LBM}}$$

EA levels were classified as high (> 40 kcal/kg LBM^/^day); optimal (≥ 40 kcal/kg LBM^/^day); subclinical (30–40 kcal/kg LBM^/^day) and clinical (< 30 kcal/kg LBM^/^day) [29].

### EI

Participants reported EI and EEE on the same days, over 7 consecutive days within two weeks of the main competition of the 2019/2020 season, before the athlete began to taper for the event. Oral and written instructions were given to the participants to explain how to weigh and record all food and drink consumed. Participants weighed their food intake using a digital kitchen scales (Camry Scale Store, California, USA). The food records were analysed using pictures taken by the participants via their mobile phones, using the weighing scales as a reference measure, in conjunction with a written food diary. Nutritional status was assessed by a trained sports nutritionist using the Nutritics software (Nutritics LTD, Ireland). The possibility of misreporting was assessed by calculating the mean reported EI to the predicted RMR ratio (rEI: RMR) [30] on an individual level:

$$\begin{gathered} {\text{EI:}}{\text{BMR}} > {\text{PAL}} \times \exp \left[ {{\text{S}}{{\text{D}}_{\min }} \times \frac{{\left( {S/100} \right)}}{\sqrt n }} \right] \hfill \\ S = \sqrt {\frac{{{\text{CV}}_{{\text{wEI}}}^2}}{d} + {\text{CV}}_{{\text{wB}}}^2 + {\text{CV}}_{{\text{tP}}}^2} \hfill \\ \end{gathered}$$

SD_min_ = − 2; SD_max_ was + 2 (95% confidence limits); CV_wEI_ = within-subject variation in energy intake (23%); CV^2^_wB_ = within-subject RMR accuracy (8.5%); CV2tP = within-subject variation in physical activity level (PAL) (15%); d = days of dietary intake. A PAL value of 1.85 for moderately active or active lifestyles was selected.

### EEE

EEE was assessed using a Polar H10 HR monitor (Polar Electro, Kempele, Finland) and recorded in the participant’s training log. Kilocalories expended were estimated based on the HR and $\dot{V}\text{O}$_2_ values extrapolated from the athlete’s $\dot{V}\text{O}$_2max_ test, using the Weir equation (per minute):

$$\left( {3.94\left( {\dot V{{\text{O}}_2}} \right) + \, 1.1 \, \left( {{\text{VC}}{{\text{O}}_2}} \right)} \right)$$

and multiplied by the number of minutes of exercise performed in each bout.

### Anthropometric assessment

Following the recording of EI and EEE, prior to the main competition, participants attended the lab in a fasted state between 8 and 10 am to assess anthropometrics, RMR and have fasted blood samples taken. Participants were instructed not to exercise in the 24 h leading up to the testing session and to maintain their normal diet. Height (cm) was measured, without shoes, to the nearest 0.1 cm using a Seca stadiometer (Seca, California, USA). Body mass (kg) was measured in minimal light clothing (i.e., shorts) to the nearest 0.01 kg using a Seca weighing scale (Seca, California, USA). Body mass index (BMI) was calculated as body mass (kg) divided by height (m^2^). LBM was assessed via skinfold measures at 7 sites (biceps, triceps, subscapular, iliac crest, supraspinale, abdominal, front thigh and medial calf) using International Society for the Advancement of Kinanthropometry (ISAK) techniques previously described by Norton [31]. Measurements were taken by a Level I ISAK accredited anthropometrist with a technical error of measurement of ≤ 3.0% using a Harpenden calliper (Baty International, Burgess Hill, England). Body density values were calculated using the male-specific equation from Jackson and Pollock [32]

$${\text{Body Density }}\left( D \right) = \, 1.112 - \left( {0.00043499 \times {\text{sum of skinfolds}}} \right) + \left( {0.00000055 \times {\text{square of the sum of skinfold sites}}} \right) - \left( {0.00028826 \times {\text{age}}} \right).$$

The body density equations were converted into a fat percentage, by using the Siri equation: %BF = ((4.95/D) − 4.50) × 100, for the analysis.

### RMR assessment

Indirect calorimetry using a canopy hood system was used (Moxus, AEI Technologies, Inc., Pittsburgh, Pennsylvania), which was calibrated before each test according to standards. Participants rested lying down for 15 min In a quiet dimly lit room before the measurements began. Oxygen consumption ($\dot{V}\text{O}$_2_) and carbon dioxide production ($\dot{V}\text{CO}$_2_) were assessed over 30 min. The last 20 min of measurement were used to assess RMR. Measured RMR was assessed using the Weir (1990) equation (per day):

$$\left( {3.94 \, \left( {\dot V{{\text{O}}_2}} \right) + \, 1.1 \, \left( {\dot V{\text{C}}{{\text{O}}_2}} \right)} \right) \times 1440.$$

The Cunningham [33] equation was used to calculate the ratio between measured RMR and predicted RMR [34]. The lowest obtained HR during the measurement of RMR was recorded using a Polar V800 HR monitor.

### Haematological analysis

Morning fasted intravenous blood samples were drawn from the cephalic vein of the participant, by a trained phlebotomist, whilst seated. Blood was drawn into a 10 ml BD Vacutainer and processed to serum. Blood samples were analysed for triiodothyronine (T_3_), total testosterone, cortisol, leptin, insulin and IGF-1 levels. Blood was also analysed for reference markers of bone formation (P1NP—procollagen type 1 N-terminal propeptide) and resorption (β-CTX—cross-linked C-telopeptide of type I collagen) (International Osteporosis Foundation and International Federation of Clinical Chemistry, 2016). Additionally, a haematology analyser (Coulter A^C^T diff™; Beckman Coulter, USA) was used to measure red blood cell (RBC) count, haemoglobin status and white blood cell count.

### Biochemical analysis

β-CTX, P1NP, T_3_, testosterone, cortisol, leptin, insulin and IGF-1 were measured in duplicate using an enzyme-linked immunosorbent assay (ELISA) (Assay Genie, Dublin, Ireland) on a BioTek^®^ ELx800™ microplate reader (Santa Clara, USA). The intra-assay coefficient of variation (CV) for β-CTX, P1NP, T_3_, testosterone, cortisol, insulin, leptin and IGF-1 was < 8%.

### $\dot{V}O$_2max_

Three hours after the consumption of a standardised breakfast, $\dot{\text{V}}\text{O}$_2max_ was determined through an incremental test to exhaustion: triathletes on a stationary bike (WattBike Pro Trainer, Wattbike Ltd, Nottingham, UK) or treadmill (HP Saturn, Munich, Germany), runners on a treadmill as congruence between the mode of ergometer and the sport activity has been shown to improve the validity of the test results [35]. The cycle ergometer protocol began with a 10-min warm-up of cycling at a power output corresponding to 3 W/kg and increased by 25 W/min until voluntary exhaustion or failure to maintain a cadence ≥ 70 RPM. The treadmill protocol began with a 10-min warm-up at 8–10 km/h with 1% gradient to represent air resistance [36]. Speed was increased by 1 km/h every 3 min until volitional exhaustion. $\dot{V}\text{O}$_2_ was measured using Moxus metabolic cart (AEI Technologies, Inc., Pittsburgh, Pennsylvania) with a mixing chamber and 30 s sampling time using a two-way T-shape non-rebreathing valve and a reusable nose clip series 9015 (Hans Rudolph, Kansas, MO, USA). HR and $\dot{V}\text{O}$_2,_ $\dot{V}\text{CO}$_2_, ventilatory equivalent (VE) and respiratory exchange ratio (RER) were monitored every 30 s throughout the test. All systems were calibrated before and after each testing session.

### Statistical analysis

Statistical analysis was completed using SPSS version 27.0 (SPSS, Inc., Chicago, IL, USA) with the alpha level set at *P* ≤ 0.05. Descriptive methods were used to compile participant characteristics. The normality of data was assessed using the Shapiro–Wilks test. Wilcoxon Signed Ranks was used for data not normally distributed. The ratio between P1NP and β-CTX was calculated to quantify bone turnover as used previously [37]. The participants’ mean endocrine and bone metabolism markers were compared to the normal population mean using 1- sample *t* tests. To assess any possible relationship between EA, macronutrients and blood markers outside of the reference range, Pearson bivariate correlations and Spearman’s correlation coefficients were conducted for normally and abnormally distributed data, respectively.

## Results

Sixteen participants began the study, three were eliminated for lack of compliance with study procedures, yielding a total of 13 participants (*n* = 10 runners, *n* = 3 triathletes). Demographic information, body composition data, energy availability components and macronutrient intakes are reported in Table 1. EA was reported as clinical (< 30 kcal/kg LBM/day) in 10 (77%) of athletes. Based on the athletes’ training logs, each athlete expended 918 ± 247 kcal through exercise, adjusted for RMR, per week. Mean carbohydrate intake was below the recommended intake of 7-12 g/kg/day [38]for endurance athletes in 12 out of 13 of participants (92%). Three out of 13 participants (23%) consumed the recommended amounts of protein per day (1.2–1.6 g/kg) [39], 10 out of 13 participants (77%) consumed above the recommended intake (> 1.6 g/kg) for endurance athletes. Two out of 13 participants (15%) consumed more than the recommended amount (20–30%) [40] of dietary fats, and 11 out of 13 participants (85%) reported daily dietary fat intakes within the recommended intake. Three out of 13 participants (23%) presented with suppressed RMR.

**Table 1:** Physical characteristics, dietary intake and measurements related to the energy intake and expenditure of participants (*n* = 13)

Variable	(Mean ± SD)	95% CI
Age (years)	34 ± 5	31–37
Height (cm)	176 ± 6	172–180
Body mass (kg)	76 ± 12	68–83
Fat mass (kg)	9.3 ± 4.4	6.6–12.1
BMI (kg/m^2^)	24 ± 2.4	20.5–25.1
RMR ratio (measured/predicted)	0.98 ± 0.2	0.86–1.1
Resting HR (bpm)	50.7 ± 5.4	47.4–54.1
Training (Hours/week)	12.3 ± 2.5	10.3–15.7
$\dot{V}\text{O}$_2max_ (ml/kg/min)	61.8 ± 4.5	59.0–64.5
EA (kcal/kg LBM/day)	27.3 ± 3.7	23.8–30.8
EI (kcal/day)	2723.3 ± 327.6	2412.1–3034.5
Carbohydrates (g/kg)	4.8 ± 1.1	4.2–5.4
Fats (g/kg)	1.1 ± 0.2	0.98–1.23
Protein (g/kg)	1.75 ± 0.37	1.53–1.97

Values expressed as means ± standard deviation (SD), *CI* confidence interval, RMR resting metabolic rate, *HR* heart rate

Reproductive, metabolic, satiety hormone concentrations, markers of bone turnover concentrations and haematological parameters are presented in Table 2. Mean levels of testosterone, cortisol, T_3_, P1NP were similar to normal male population means, with no participants displaying any values outside the normal ranges [41–43]. Mean RBC level was significantly lower than the population norm of 4.6–5.7million/mcL [44], with 8 out of 13 (62%) participants presenting with low RBC count. Mean haemoglobin level was significantly different from the normal mean of the male population (13.5–18 g/dL [44]), with 6 out of 13 (46%) participants displaying low haemoglobin levels. Four out of 13 participants (31%) were identified with low haematocrit concentrations, with mean haematocrit level significantly different than the normal level for healthy males (0.430–0.510 g/dL) [44]. Mean level of insulin was below the reference range (25–70 pmol/L) in 4 out of 13 participants (31%). Mean level of IGF-1 was lower than the reference range (100-400 ng/ml) in 3 out of 13 participants (23%). Mean level of leptin was significantly lower than normal male population means with 8 out of 13 (62%) participants displaying levels lower than the minimum level of 2000 pg/ml. Mean level of β-CTX was significantly higher than normal male population mean with 11 out of 13 (85%) participants displaying higher levels than the upper recommended level of 830 pg/ml. Twelve out of 13 (92%) participants presented with PINP:β-CTX ratio < 100, which was significantly lower than the normal population mean [45].

**Table 2:** Reproductive, metabolic, satiety hormone concentrations, markers of bone turnover concentrations and haematological parameters in highly trained male endurance athletes (*n* = 13)

Biochemical marker	Normal range	Mean ± SD	95% CI	P value
Testosterone ng/dL	221–847 [41]	609 ± 164	510–708	0.063
Cortisol ng/ml	63–250 ng/ml [42]	169 ± 34	148–190	0.092
T_3_ pg/ml	800–2100 [79]	1420 ± 345	1212–1629	0.383
Leptin pg/ml	2000–6000 [69]	1759 ± 1112	1087–2431	< 0.001
P1NP ng/ml	15–80 [43]	52 ± 0.4	51–52	< 0.34
β-CTX pg/ml	120–830 [75]	2161 ± 1363	1337–2985	< 0.001
Insulin pmol/L	25–70 [73]	29.44 ± 21.2	15.6–41.7	0.01
IGF-1 ng/ml	100–400 [66]	208 ± 24	194–223	< 0.001
PINP: β-CTX	< 100 [45]	45 ± 55	− 9.5–42.7	0.004
White blood cells × 10^9^/L				
Runners	3.9–10.2 [80]	4.15 ± 0.9	3.7–9.6	0.361
Triathletes	3.5–9.9 [80]	4.15 ± 0.9	3.3–9.4	0.311
Red blood cells (million/mcL)	4.6–5.7 [44]	4.44 ± 0.45	4.16–4.7	< 0.001
Haemoglobin (g/dL)	13.5–18 [44]	13 ± 1.2	12.2–13.7	< 0.001
Haematocrit (g/dL)	0.430–0.510 [44]	0.39 ± 0.29	0.37–0.4	< 0.001
Lymphocytes (%)	20–40 [44]	35.9 ± 7.1	31.6–40.2	0.110
Monocytes (%)	2–8 [44]	4.96 ± 1.6	3.9–5.9	0.932

Values expressed as means ± standard deviation (SD); CI = Confidence Interval; T_3_ = triiodothyronine; P1NP = procollagen type 1 N-terminal propeptide; β-CTX = cross-linked C-telopeptide of type I collagen; IGF-1 = Insulin Like Growth Factor 1; MCHC = mean corpuscular haemoglobin concentration. One-sample *t* test using the group means and the mean of the normal range for males were conducted to generate *P* value

### Correlations

Significant correlations were reported between EA and carbohydrate intake (*r* = 0.718, *P* = 0.006) and lean mass (*r* = − 0.636, *P* = 0.019). No other significant correlations between EA, macronutrients or blood markers were identified.

## Discussion

This cross sectional study was designed to measure the incidence of LEA, as defined by the evidence-based female thresholds [29], in highly trained male endurance athletes. In addition to this, macronutrient intake, hormonal status, and blood measures were also compared to the desired and normal levels. The primary finding of this study was that 76.9% (*n* = 10) of participants presented with LEA. Furthermore, mean levels of blood sugar regulating hormone insulin, cell growth hormone IGF-1, oxygen carrying components haemoglobin, haematocrit, RBC, and satiety hormone leptin were significantly lower than their respective reference ranges for males (Table 2). Moreover, mean level of β-CTX was significantly higher than normal male population mean in 11 out of 13 participants, indicating elevated bone resorption rates. Additionally, PINP: β-CTX ratio was < 100 in 12 out of 13 participants, also indicating elevated bone resorption [45].

### Energy availability (EA)

Mean EA was < 30 kcal/kg LBM/day in 76.9% of participants which, according to Melin et al., [29], is clinically relevant and potentially symptomatic. This incidence of LEA is slightly higher than reported in previous similar studies which observed prevalence rates of 66% and 61%, respectively [7, 46] in highly trained male endurance athletes. A number of study design differences exist between the current study and the aforementioned studies Firstly, Lane et al., [7] assessed EI for 2 weekdays and 2 weekend days and Klein et al., [8] recorded EI for 2 weekdays and 1 weekend day which may not have captured dietary intake on various training days [47], whereas the current study recorded dietary intake over 7 days. Furthermore, the wide age range of participants (21–70 years) recruited renders it difficult to draw accurate comparisons to the current cohort, aged 19–40 years. In particular, the natural decline in bone [48] and muscle mass [49] may result in an overestimation of EA status. Similar to the current study, Jurov et al., [46] assessed dietary intake and exercise energy expenditure over 7 consecutive days. However, although the authors state that HR monitoring was used to measure calories expended during exercise, it is unclear what method was used. It is also possible that the EA level was overestimated by Jurov et al., [46]. Unlike the current study which used skinfold measures, Jurov et al., [46] and Klein et al., [8] assessed body composition via bioelectrical impedance which has been shown to overestimate body fat percentage in lean individuals [50], thus underestimating LBM resulting in a higher EA score. Until a standardised approach is established to accurately measure EA, discrepancies between studies will continue to remain.

### Macronutrient intake

Twelve out of 13 participants (93%) did not consume the recommended daily intake of 7–12 g/kg of carbohydrates for endurance athletes [51], with lower carbohydrate intake strongly associated with lower EA (*r*^2^ = 51.5%). When exercising at high intensities or for extended durations, carbohydrate becomes the primary energy source, and thus inadequate carbohydrate availability may be a limiting factor for performance [52]. Carbohydrate restriction is a common method used to reduce body fat in athletic cohorts [53]. Furthermore, it has been suggested that residual fatigue from training may decrease appetite and thus reduce carbohydrate intake [54]. Regardless of the reasons, educating endurance athletes on the importance of appropriate carbohydrate intake and navigating the obstacles they may face when consuming carbohydrates is critical.

Dietary protein is essential to repair damaged muscle fibres, which provides the basis for many training adaptations [55]. Protein intake in this study was in line with previous research on endurance athletes [56] with 10 of the 13 athletes (77%) exceeding the recommended amount of 1.2–1.6 g/kg for endurance athletes [39]. However, emerging evidence has suggested that protein intake of > 1.6 g/kg would be more beneficial to endurance athletes as the nitrogen balance technique used in the estimation of protein requirements in previous studies has limitations, such as overestimating nitrogen intake, which would ultimately result in an underestimation of protein requirements [57]. Moreover, Gillen et al., [57] concluded that endurance athletes consuming suboptimal carbohydrate intake require approximately 1.83 g/kg of protein on a training day in order to replace amino acid oxidative losses incurred during exercise, and to provide the necessary substrates for postexercise protein synthesis. Therefore, considering the carbohydrate intake of these participants, elevated protein levels may be appropriate.

Mean fat intake was 1.1 ± 0.2 g/kg, with all participants reporting at least 20% calorie contribution from fat, in accordance with previous studies [6]. Two athletes exceeded the recommended intake of 20–30% calorie intake from dietary fats [40], which may have contributed to the underconsumption of carbohydrates.

### Body mass index and resting metabolic rate

Athletes participating in sports which emphasise leanness, such as endurance events, with BMI ≤ 17.5 kg/m^2^ are considered at high-risk for low BMD [58]. No participants in the current study had a BMI approaching this low level. RMR represents the energy required for basic physiological functioning [59] and appears to be suppressed by LEA [3]. RMR_ratio_ (measured RMR/predicted RMR) has been suggested as a surrogate marker for LEA, with an expected normal range of 0.9–1.1[33]. Moreover, a low RMR_ratio_ (< 0.90) is proposed to represent LEA [60]. Four out of 13 athletes (31%) presented with a low RMR_ratio_ in the current study, with 3 of those presenting with LEA. The mean RMR_ratio_ was 0.98 ± 0.2 which is surprising due 10 participants being in a state of LEA. However, it still remains unclear whether the RMR_ratio_ range of 0.90–1.10 is appropriate for males and perhaps the EA level was not low enough to exhibit suppressed RMR in the entire group [2].

### Blood measures

Mean RBC, haemoglobin and haematocrit levels of participants were significantly lower from the normal mean of the male population. Eight out of 13 participants presented with low RBC counts with 6 out of 8 of these in LEA. 6 out of 13 (46%) reported with low haemoglobin levels, with 4 of these participants in LEA. 4 out of 13 (31%) with low haematocrit concentrations, with all 4 presenting with LEA also. These results indicate that a number of these athletes may be at risk of exercise-induced anaemia [61]. This reduction may be transient and simply due to the period of high volume training causing the blood plasma volume to increase more quickly and to a greater extent than RBC volume [62]. Due to the small sample size in this study, it was difficult to determine if these results were significantly associated with EA status. However, similar levels of RBC count and haemoglobin were reported by Jurov et al., [12], when EA was deliberately reduced by 25% (mean EA 22.4 ± 6.3 kcal/kg LBM/day) in (*n* = 12) trained, well-trained and elite male endurance athletes for 14 days. Since this may affect the oxygen-carrying capacity of the blood, $\dot{V}\text{O}$_2max_ and thus negatively affect aerobic performance, further investigation is warranted.

Cortisol levels were within the reference range for healthy males (63–250 ng/ml) for all participants [42]. Due to the interplay between exercise stress and cortisol levels, the interpretation of cortisol as a marker of LEA should be approached with caution, particularly due to the large volumes of training that are undertaken by highly trained endurance athletes [63]. Cortisol levels are regulated by brain glucose availability [64], therefore extensive periods of LEA, particularly reduced carbohydrate availability, may cause cortisol to rise in order to spare glucose for brain function [64]. More in-depth research is needed to understand the effects of LEA on cortisol in male athletes.

Mean testosterone levels were within the normal range for healthy young athletic males [41], with no significant difference between mean testosterone concentrations of the participants and the normal male population levels. It could be suggested that EA was not low enough to induce clinically low testosterone levels in the current study, however, Koehler et al., [2] also reported no significant effect of LEA on testosterone levels in active males when EA was 15 kcal/kg LBM/day. It should be noted that circulating concentrations of binding hormones, such as sex hormone-binding globulin, may have reduced the bioavailability of testosterone and therefore blunted the endocrine suppression during both Koehler’s study and the present study. However, these binding hormones were not measured. Future studies should consider assessing testosterone in saliva as it is a validated method that appears to be unaffected by circulating binding proteins [65] and measuring SHBG levels.

IGF-1 concentrations were significantly below the normal range (100-400 ng/ml) [66] in 3 out of 13 participants, with 2 of these athletes presenting with LEA. This is similar to well-controlled studies in active males by Koehler et al., [2] and Papageorgiou et al., [35]. However, Koehler et al., [2] suggested the reduced baseline leptin concentrations (< 2 ng/ml) may have acted to prevent changes in IGF-1 and other endocrine axes. In the current study, 61% (*n* = 8) of athletes presented with leptin levels < 2 ng/ml, which is within the lowest quartile of the normative range (0.9–7.7 ng/ml) [67], and may have blunted the alterations in IGF-1 in response to LEA.

Leptin is an appetite-suppressing hormone, strongly associated with fat mass and appears to be reduced in situations of restricted energy intake [68]. Mean leptin levels were significantly lower than the normal population mean in males, with 8 out of 13 (62%) participants displaying levels lower than the minimum level of 2000 pg/ml [69], and 6 of these presenting with LEA. The lower leptin levels could be the result of a reduction in fat mass [70]. Data collection in the current study took place at one time point during the competition season, therefore any fluctuations in the participant’s fat mass prior to this point are unknown. However, athletes often endeavour to reduce fat mass leading up to a competition as a lower body fat percentage is considered advantageous during competition. Furthermore, mean body fat % was lower in the current study (11.2%) than in previous research (16.8%) [71], therefore it is plausible that a reduction in fat mass was partially responsible for the lower concentrations of leptin. The lower concentrations of leptin may pose a deleterious effect on bone metabolism as when leptin is reduced for prolonged periods, it may facilitate the rise in cortisol levels that has been shown to inhibit bone formation marker P1NP [72]. Furthermore, significantly lower insulin levels were identified in 3 out of 13 (23%) participants [73]. Both leptin and insulin are linked to adiposity and when energy balance shifts negatively, leptin and insulin levels will decrease and send signals to the brain about the quantity of fat mass, its distribution and fluctuations in metabolic status [74]. As previously mentioned, reductions in fat mass resulting in low body fat % may have occurred in these athletes in the weeks or months leading up to this study, stimulating the reductions in leptin and insulin.

An interesting finding of this study was that mean β-CTX levels were significantly above the normative range in 11 out of 13 athletes, indicating increased bone absorption [75]. Furthermore, 9 of these athletes were in LEA. Similar findings have been reported in healthy, active, non-sporting females [1, 37] but not in active males [37]. Previous research has shown that chronic, exhaustive, endurance exercise induces a transient stimulation of bone resorption in the subsequent days following exercise [76], as the athletes in the present study took part in endurance-based training for 12.3 ± 2.5 h per week, it suffices to say that these athletes may have been continuously under threat of elevated bone resorption. Recent research has also shown that carbohydrate availability, regardless of EA status, may influence β-CTX concentrations in endurance-trained males [77]. Hammond et al., [77] found that carbohydrate intakes of ~ 12 g/kg/day in comparison to ~ 3 g/kg/day attenuated circulating β-CTX concentrations in male endurance athletes. The mean carbohydrate intake of the present study was 4.8 ± 1.06 g/kg/day which may be too low to abate the elevation in β-CTX concentrations. Due to the small sample size in this study, correlations could not be drawn between β-CTX concentrations and EA status, however future research should endeavour to investigate this further.

Mean concentrations of P1NP were within the reference range for males [43]. However, the mean P1NP: β-CTX ratio was < 100, indicating a state of bone resorption [45]. Further to this, the reduced IGF-1 levels observed is considered an additional threat to bone health [78]. Contrary to these findings, previous research in healthy, active, non-sporting females found that LEA significantly reduced markers of bone formation [1]. However, different markers of bone formation were used by Ihle and Loucks [1] (NTX) in addition to different sampling methods (plasma and urine). Therefore, it is difficult to draw direct comparisons. However, Papageorgiou et al., [37] also investigated the effect of LEA (15 kcal/kg LBM/day) on P1NP in active males and females over 5 days. Results showed that P1NP was significantly reduced in healthy, active, non-sporting females when EA was 15 kcal/kg LBM/day. However, similar to the current study, significant effects were not found in healthy, active, non-sporting males.

It was suggested by the authors that active females may be more sensitive to the effects of LEA on markers of bone formation than males and that perhaps more extended exposure to LEA is needed to elicit effects in males. However, based on the limited number of studies, further longitudinal, strictly controlled research on highly trained male endurance athletes is needed to validate this theory.

### Practical applications

This research is the first of its kind in Irish endurance male athletes. The EA status and inadequate carbohydrate intake in these athletes is of concern, indicating a need for nutritional education specifically related to EA and EI to support the development of optimal health and performance. Due to the small sample size within this study, the reduced mean levels of insulin, IGF-1, leptin and oxygen carrying components haemoglobin, haematocrit, RBC, and the elevated β-CTX levels could not be definitively associated with EA status, however future large scale studies may identify such relationships. However, the bone status of these highly trained male endurance athletes, regardless of EA status, is a concern as elevated levels of bone resorption are evident. An industry-led bone health intervention programme should be considered to support optimal bone health in this population. Finally, it still remains unclear whether the RMR_ratio_ range of 0.90–1.10 is appropriate for males and whether the LEA threshold of < 30 kcal/kg LBM/day is low enough to elicit metabolic and endocrine disturbances.

## Conclusion

This study demonstrates that LEA (< 30 kcal/kg LBM/day) is prevalent in highly trained Irish male endurance athletes. However, the data obtained represents a snapshot assessment of one week during the competition season and cannot be generalised across each stage of a season. Moreover, due to the small sample size, associated effects of on endocrine function or bone metabolism could not be established. Irrespective of EA, these athletes are also at risk of elevated levels of β-CTX, and reduced leptin levels which, when positively associated with P1NP and inversely associated with cortisol levels, may lead to impairments in bone health. Furthermore, highly trained endurance athletes may be at risk of reduced oxygen-carrying capacity due to suboptimal RBC count, haemoglobin levels and haematocrit concentrations, future research should include sub-maximal and maximal endurance tests to substantiate these findings. Because these metabolic concentrations fluctuate over time and may be linked to long-term health consequences, further larger-scale, longitudinal research is needed to investigate the mechanisms behind the various endocrine responses to EA.
